# Supplementary material for: Learning curve and surgical outcome of robotic assisted colorectal surgery with ERAS program
Source: Sci Rep. 2022 Nov 29;12:20566. doi: 10.1038/s41598-022-24665-w (PMC9709162; doi:10.1038/s41598-022-24665-w)
Supplement: Supplementary file 1 — Supplementary Information 1. [file 41598_2022_24665_MOESM1_ESM.zip › LSC Raw data-Ñ[▒K/118/H_ñw╝╨░O▒Kñσ.pdf]

正式摘要(電子簽章：0000002114831?6)

W55 / W55 ☐ 英文標題(注意:無英文姓名-請補病人基本檔!) ☐【讀取】憑證

臺中榮民總醫院

出院病歷摘要

轉入號： 轉出號：

[redacted]

(8)流水編號	
(9)入院日期	110/02/24 (2021) 直腸外科 W55 - 111
(10)轉科日期	
(11)出院日期	110/03/03 (2021) 住院天數計 7 日

診斷

- 入院：1. Descending colon tumor
  2.Adenocarcinoma of prostate, cT3aN1M2 (bone) , s/p channel TURP on 2018-6-15 now under hormone therapy (Zoladex, Casodex)
  3. Hypertension
  4. CAD
  5. Inguinal hernia s/p hernioplasty
- 出院：1. Descending colon tumor s/p laparoscope left hemicolectomy on 2021/2/25
  2.Adenocarcinoma of prostate, cT3aN1M2 (bone) , s/p channel TURP on 2018-6-15 now under hormone therapy (Zoladex, Casodex)
  3. Hypertension
  4. CAD
  5. Inguinal hernia s/p hernioplasty

主訴

- S colon tumor noted for one month during regular follow up

病史

- This is a 81 year old patient with history of
  1.Adenocarcinoma of prostate, cT3aN1M2 (bone) , s/p channel TURP on 2018-6-15 now under hormone therapy (Zoladex, Casodex)
  2. Hypertension
  3. CAD
  4. Inguinal hernia s/p hernioplasty
  the patient underwent regular follow up at GU OPD and underwent hormone therapy. CT on 2021/2/2 showed S colon ca with interssusception, lung CT was done and showed no metastasis. Colonoscope was done and showed 60cm FAV tumor s/p biopsy. Under the impression of S colon ca, the patient was admitted for further treatment.

體檢發現

- CEA 2.0(2020/9)-
  COS: TUMOR 40 CM FAV
  2/8 LUNG CT NEG. ABD CT
  - General appearance :
    - easy looking , conscious clear , without cardiopulmonary distress
    - Vital signs自動帶入date 體溫(BT) 脈搏(P) 呼吸(R) 血壓(BP) 身高(BH) 體重(BW) BMI
      20210224 36.3℃ 80次/分 18次/分 136/67mmHg 168.3cm 58.3kg 20.6
    - Vital signs: B.P. 136/94 mmHg , P.R. 108 /min , R.R. 18 /min , B.T. 37 ℃
  - HEENT :
    - Eyes: no anemic conjunctiva, no icteric sclera, normal EOM
    - ENT: no otorrhea, no rhinorrhea, no erythematous mucosa, no injected tonsil, no deformity
  - Neck: supple, freely movable, trachea midline,no deformity, no engorged jugular vein , no palpable thyroid or lymph node enlargement
  - Chest:
    - Inspection: Grossly normal, symmetric and fully expansion, no accessory muscle used, no operation scar, no spider angiomata, no gynecomastia
    - Palpation: no palpable mass or nodule, no subcutaneous emphysema, no tenderness
    - Ausculation: clear breathing sound
  - Heart:
    - Percussion: normal heart size
    - Ausculation: regular heart beat, normal S1, S2, no gallop, no murmur
  - Abdomen:
    - Inspection: in
      - flat contour, symetrically, no scar, no stria, no dilated veins, no rashes
    - Ausculation: normoactive bowel sound, no abdominal bruits
    - palpation: soft, no muscle guarding or rigidity, no local tenderness, no rebounding pain, no palpable mass, no palpable pulsation
    - Percussion: no shifting dulllness, no hepatomegaly, no splenomagaly
  -

Peripheral pulsation :	CA	BA	RA	FA	PA	PTA	DPA
Right side :	++	++	++	++	++	++	++/+++
Left side :	++	++	++	++	++	++	++/+++

  - Genito-anus: no tenderness or palpable mass lesion or rectal shelf, normal external genitalia, normal anal tone, brown stool

CRS CONDITION

-
  - Lung

手術日期及方法

- 手術日期及方法20210225 Laparoscopic Anterior resection and anastomosis - malignant

住院治療經過

- After admission, laparoscope left hemicolectomy was done smoothly on 2021/2/25. The patient on ERAS protocal and try bland one diet on 02/26, then BLAND II diet on 02/27 without abdomen discomfort. Penrose was removed on 03/02 then discharged the aptient on 03/03 with stable condition.

合併症

一般檢查

- 累積報告：

DATE	TIME	ALB	TP	BIL.T	BIL.D	ALKP	AST	ALT	LDH	CHOL	TG	HDL-C	LDL	UA	NH3	AMY
1100225	2045	2.9		0.48			22	14								
1100301	1010			0.31	0.11		26	10								

DATE	TIME	WBC	RBC	HGB	HCT	MCV	PLT	BLST	NEUT	BAND	SEG	LYM	MON	EOS	BAS
1100225	2044	22790		9.7			350		89.8			6.9	3.1	0.0	0.2
1100301	0951	9990		8.7	26.5		304		83.4			11.6	3.4	1.1	0.5

DATE	TIME	NA	K	CL	CA	BUN	CREAT	Mg	P	CRP	ESR	PCT	LIPASE	LACTATE
1100225	2045	142	3.1			12	0.99							
1100301	1010	138	3.4			10	1.09							

- 放射線報告：檢驗項目 **:CHEST PA VIEW** 報告日期 **:1100209**
  主 訴：

- Result:
  N
  Finding:
  1. Tortuous and atherosclerosis of thoracic aorta.
  2. Slight widening of mediastinum.
  3. No cardiomegaly.
  4. Slight increase infiltration of bil. lungs, in favor of chronic inflammation.
  5. Scalloping of bil. hemidiaphragm.
  6. DJD change of T and L spine.
  Impression:
  As above description.

  檢驗項目 **:CT, LUNG** 報告日期 **:1100210**
  主 訴：
  Result:
  Y
  Finding:
  CT scan of chest, hila and trachea with contrast enhancement:
  1. No obvious abnormal nodular lesion in both lungs.
  2. Mild increasing interlobular septal thickening in bilateral basal peripheral lungs are noted. Suggest F/U to differentiate from usual interstitial pneumonia (UIP) and lymphangitis carcinomatosis.
  3. No significant lymphadenopathy in the mediastinum and bilateral hilar regions.
  4. R/O several small cysts in liver. Suggest F/U.
  5. An osteoblastic lesion in T8 vertebral body is noted. Suggest F/U.
  Impression:
  No obvious abnormal nodular lesion in both lungs, F/U.

  檢驗項目 **:CT ABDOMEN-SURVEY** 報告日期 **:1100203**
  主 訴：
  Result:
  Y
  Finding:
  MDCT FROM DIAPHRAGM TO PUBIC SYMPHYSIS WITH THREE PHASES AND SLICE THICKNESS 4MM SHOWED:
  1. UNDER PAST HX OF CAP S/P TURP BX PROVED.
  PROSTATE GLAND IS NOT ENLARGED.
  WELL DISTENDED U-BLADDER IS ALSO NOTED.
  2. MILD FATTY LIVER IS FOUND. PANCREAS AND SPLEEN ARE NOT UNUSUAL.
  ATROPHIC CHANGE OF PANCREAS IS ALSO NOTED, MAYBE DUE TO CHRONIC PANCREATITIS. THE SPLEEN IS NOT UNUSUAL.
  SUSPICION OF HAVING LESION IN DISTAL S-COLON (701#94) WITH INTUSSUSCEPTION (#91-95). MAYBE DUE TO MALIGNANT PROCESS SUCH AS S-COLON CA. FECAL MATERIALS ARE IMPACTED IN D-COLON.
  3. THE BILATERAL ADRENAL GLANDS ARE NOT UNUSUAL.
  NORMAL FUNCTION OF BILATERAL KIDNEYS AND THEIR PELVOCALYCEAL SYSTEMS ARE NOT UNUSUAL.
  4. ATHEROSCLEROSIS OF ABDOMINAL AORTA AND PELVIC ARTERIES ARE NOTED.
  5. NO LYMPHADENOPATHY IN RETROPERITONEUM AND BILATERAL PELVIS BUT WITH NON-SIGNIFICANT LYMPHADENOPATHIES IN BILATERAL INGUINAL AREAS.
  6. THERE ARE DEGENERATIVE JOINT DISEASES FOUND IN THORACO-LUMBAR SPINE.
  7. THE BILATERAL LOWER LUNGS ARE CLEAR.
  Impression:
  1. SUSPECTED S-COLON CA WITH INTUSSUSCEPTION
  2. CAP S/P TURP BX PROVED WITH PROSTATE SIZE NOT ENLARGED

特殊檢查

病理報告

- 活體組織切片病理報告 手術日期：**110/02/08** 病理號：**S1004961**

  診斷醫師:李欣倪 報告日期:110/02/17
  Pathologic diagnosis:
  Sigmoid colon, endoscopic biopsy --- Necrotic tissue. See comment.
  Ancillary study for diagnosis:
  IHC stains for AE1/AE3 (highlight epithelia) and CMV (-) done.
  Gross description:
  The specimen consists of a piece of tan soft tissue, 0.2x0.2x0.2 cm.
  All for section.
  Microscopic description:
  Section shows necrotic tissue with acute inflammatory cells infiltration.
  Comment:
  The specimen might not be fully representative. Only necrotic tissue and inflammatory cells are found. Further clinical correlation and re-sampling, if clinically indicated, are suggested.
  #T-59300_2 #M-54000_2 2 1340 00000F

其它

出院時情況

- 15.計劃性出院

出院指示

藥名	劑量	劑量單位	用法	頻次	數量	數量單位	現況	天數	單位
Spironolactone-25	1	TAB	PO	BID	14	TAB	TH	7	天
(口)Rosis tab 40mg	1	TAB	PO	QD	7	TAB	TH	7	天

出院計劃

主治醫師 2062F 蔣鋒帆 / 住院醫師 2325G 劉仕梯
正式摘要(電子簽章：**000000211482176)**

W55 / W55

doctorRank:B
